# EGF promotes PKM2 *O*-GlcNAcylation by stimulating *O*-GlcNAc transferase phosphorylation at Y976 and their subsequent association

**DOI:** 10.1016/j.jbc.2022.102340

**Published:** 2022-08-03

**Authors:** Yang Wang, Hengyao Shu, Jia Liu, Xin Jin, Lihua Wang, Yanzhao Qu, Mingjie Xia, Pinghui Peng, Yunpeng Feng, Min Wei

**Affiliations:** Key Laboratory of Molecular Epigenetics of the Ministry of Education (MOE), Northeast Normal University, Changchun, Jilin, China

**Keywords:** epidermal growth factor, *O*-GlcNAcylation, *O*-GlcNAc transferase, pyruvate kinase M2, phosphorylation, phosphotyrosine-binding protein, ACN, acetonitrile, BLI, biolayer interferometry, EGF, epidermal growth factor, EGFR, EGF receptor, IP, immunoprecipitation, MS, mass spectrometry, OGA, *O*-GlcNAcase, OGT, *O*-GlcNAc transferase, PKM2, pyruvate kinase M2, PTM, posttranslational modification, TFA, trifluoroacetic acid, WB, western blotting

## Abstract

Epidermal growth factor (EGF) is one of the most well-characterized growth factors and plays a crucial role in cell proliferation and differentiation. Its receptor EGFR has been extensively explored as a therapeutic target against multiple types of cancers, such as lung cancer and glioblastoma. Recent studies have established a connection between deregulated EGF signaling and metabolic reprogramming, especially rewiring in aerobic glycolysis, which is also known as the Warburg effect and recognized as a hallmark in cancer. Pyruvate kinase M2 (PKM2) is a rate-limiting enzyme controlling the final step of glycolysis and serves as a major regulator of the Warburg effect. We previously showed that PKM2 T405/S406 *O*-GlcNAcylation, a critical mark important for PKM2 detetramerization and activity, was markedly upregulated by EGF. However, the mechanism by which EGF regulates PKM2 *O*-GlcNAcylation still remains uncharacterized. Here, we demonstrated that EGF promoted *O*-GlcNAc transferase (OGT) binding to PKM2 by stimulating OGT Y976 phosphorylation. As a consequence, we found PKM2 *O*-GlcNAcylation and detetramerization were upregulated, leading to a significant decrease in PKM2 activity. Moreover, distinct from PKM2, we observed that the association of additional phosphotyrosine-binding proteins with OGT was also enhanced when Y976 was phosphorylated. These proteins included STAT1, STAT3, STAT5, PKCδ, and p85, which are reported to be *O*-GlcNAcylated. Together, we show EGF-dependent Y976 phosphorylation is critical for OGT-PKM2 interaction and propose that this posttranslational modification might be important for substrate selection by OGT.

Epidermal growth factor (EGF) is one of the most well-characterized growth factors and required for cell growth, proliferation, and differentiation (1). As a part of the transmembrane receptor tyrosine kinases (RTKs) family, EGF receptor (EGFR) appears to be highly expressed and constitutively activated in multiple cancer types, such as lung cancer and glioblastoma (2, 3, 4). EGFR is the first identified RTK functioning as an oncogene and found to regulate cancer development by triggering distinct pathways, including RAS/RAF/MEK/ERK, PI3K/AKT/mTOR, Src kinases, and STAT transcription factors (1). Aberrantly regulated EGFR is often associated with poor prognosis. Targeting EGFR with specific inhibitors, antibodies, or vaccines has been extensively explored and recognized as useful strategies for therapeutic treatment against cancers (1, 2, 4).

Emerging evidence revealed that EGF stimulation could trigger metabolic reprogramming in multiple types of cancer cells (5). In rapidly dividing hepatocellular carcinoma cells, EGFR activation upregulated *de novo* fatty acid synthesis to meet the demand for membrane lipids (6, 7). Activation in EGF signaling enhanced nutrient uptake, especially glucose, to support the rapid proliferation of non–small cell lung cancer cells (5, 8). More recent work has demonstrated an important role for EGF in the regulation of aerobic glycolysis, which is prominently featured in tumors as the Warburg effect (8). The activation of EGFR could turn on both PI3K-AKT and tyrosine kinase signaling to mediate the phosphorylation of several key enzymes controlling glycolysis, including hexokinase, phosphofructokinase, and pyruvate kinase (PK) (5). As a consequence, functions of these enzymes were orchestrated, leading to enhanced glucose uptake as well as metabolic reprogramming that in turn support macromolecular synthesis and NADPH production (5).

As pyruvate kinase M2 (PKM2) is the rate-limiting enzyme controlling the final step of glycolysis and serves as a major regulator of the Warburg effect in different cancer cells, the regulation of PKM2 by EGF, particularly in high EGFR–expressing cells, is thus of great importance (9, 10, 11, 12, 13). It has been reported that EGF-mediated ERK1/2 activation stimulated PKM2 phosphorylation at serine 37 in glioblastomas (10). As a consequence, the enzymatic activity of PKM2 was reduced, and PKM2 was relocated into the nucleus to promote glucose transporter GLUT1 and lactate dehydrogenase LDHA expression (10). Moreover, PKM2 was found to be acetylated by p300 in breast cancer cells upon EGF stimulation. By blocking allosteric activator FBP binding, PKM2 K433 acetylation reduced its enzymatic activity and influenced glycolysis (12). More recently, highly *O*-GlcNAcylated PKM2 was observed in a series of tumor cells and tissues (14, 15). As a unique posttranslational modification (PTM), *O*-GlcNAcylation is reversibly controlled by *O*-GlcNAc transferase (OGT) and *O*-GlcNAcase (OGA) and recognized as a “nutrient sensor” (16, 17, 18, 19). *O*-GlcNAcylation at PKM2 T405/S406, by regulating PKM2 oligomerization and function, coupled glycolysis to dynamically changing environment (14). In previous work, we also observed that *O*-GlcNAcylation level at PKM2 T405/S406 was evidently increased in response to EGF stimulation (14). However, the mechanism for the regulation of PKM2 *O*-GlcNAcylation by EGF still remains unaddressed.

In this work, we showed that EGF stimulated the phosphorylation of OGT at Y976 and in turn promoted OGT binding to PKM2. As a consequence, PKM2 *O*-GlcNAcylation and detetramerization were enhanced, leading to a significant reduction in PKM2 activity. Other than PKM2, additional phosphotyrosine-binding proteins (20, 21, 22, 23, 24, 25, 26, 27), including STAT1, STAT3, STAT5, PKCδ, and p85, which are known to be *O*-GlcNAcylated, were also preferentially recognized and associated with OGT phosphorylated at Y976. Together, we showed that EGF-dependent Y976 phosphorylation is critical for OGT-PKM2 interaction and this PTM might be important for the substrate selection of OGT.

## Results

### EGF reduces PKM2 enzymatic activity by stimulating T405/S406 *O*-GlcNAcylation

**Figure 1:**
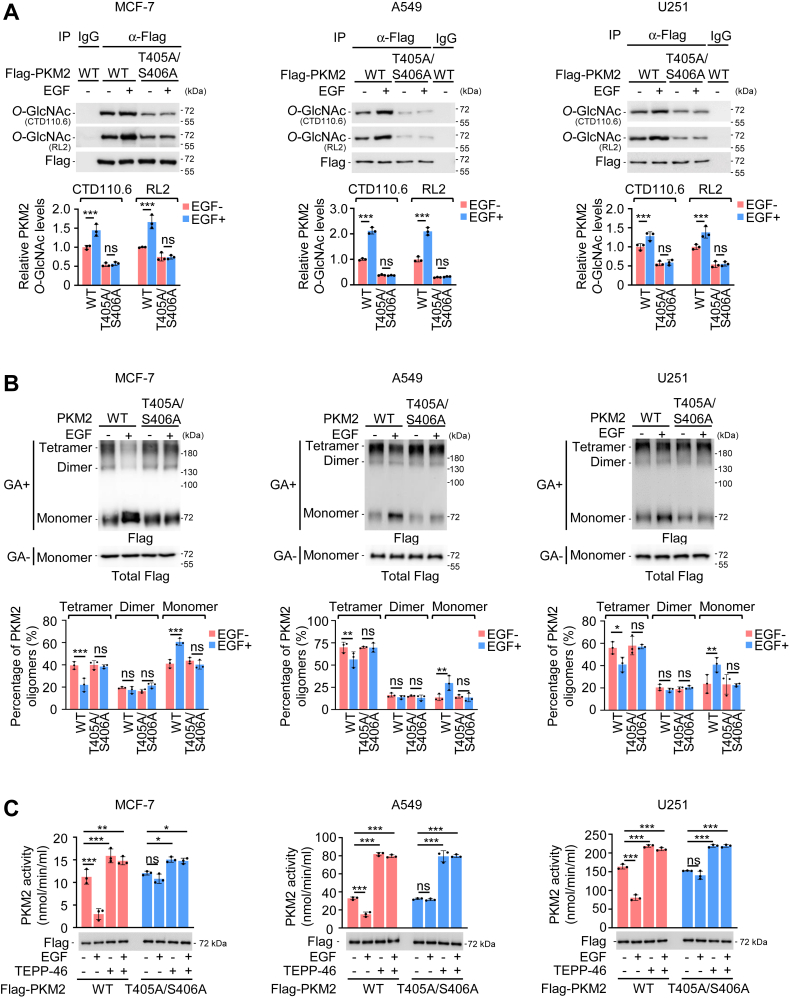
**EGF induces PKM2 detetramerization and reduced its activity.***A*, EGF upregulates PKM2 T405/S406 *O*-GlcNAcylation. (*top*) Transfection of Flag-tagged PKM2^WT^ or PKM2^T405A/S406A^ along with or without concomitant EGF treatment (100 ng/ml for 1 h) was conducted in MCF-7, A549, or U251 cells. Following Flag immunoprecipitation (IP), samples were analyzed by western blotting (WB) with antibodies against Flag and *O*-GlcNAcylation (CTD110.6 and RL2), respectively. IgG is the negative control. (*bottom*) Relative protein levels of IP. PKM2 *O*-GlcNAc was normalized to immunoprecipitated Flag-PKM2. Quantification shows mean ± SD (n = 3) with significance determined by two-way ANOVA, ∗∗∗*p* < 0.001, ns, nonsignificant. *B*, distribution of PKM2 oligomers. *(top)* MCF-7, A549, and U251 cells transfected with Flag-tagged PKM2^WT^ or PKM2^T405A/S406A^ for 24 h were incubated in the presence or absence of EGF (100 ng/ml for 1 h) and subsequently analyzed by WB. GA, glutaraldehyde. (*bottom*) Quantification of PKM2 oligomers. Tetramers, dimers, and monomers were normalized according to total Flag-PKM2 in each group. Data represent mean ± SD (n = 3) with significance determined by two-way ANOVA, ∗*p* < 0.05, ∗∗*p* < 0.01, ∗∗∗*p* <0.001, ns, nonsignificant. *C*, the effect of EGF on PKM2 activity. MCF-7, A549, and U251 cells were transfected with Flag-tagged PKM2^WT^ or PKM2^T405A/S406A^ for 24 h in the presence or absence of TEPP-46 (100 μM, 24 h). Subsequently, they were treated with or without EGF (100 ng/ml) for 1 h prior to the analysis of PKM2 enzymatic activity. Data represent mean ± SD (n = 3) with significance determined by one-way ANOVA, ∗*p* < 0.05, ∗∗*p* < 0.01, ∗∗∗*p* < 0.001, ns, nonsignificant. EGF, epidermal growth factor.

We detected upregulated PKM2 T405/S406 *O*-GlcNAcylation upon EGF stimulation in breast cancer MCF-7 cells (Fig. 1*A*) (14). EGFR is widely amplified in various cancers, such as lung cancer and glioblastoma (28, 29, 30). Deregulated EGFR signaling has a central role in driving cancer pathogenesis (28, 29, 30). Subsequently, we found this double mark was also enhanced by EGF in non–small cell lung cancer A549 and glioblastoma U251 cells (Fig. 1*A*). Once T405/S406 was mutated, PKM2 *O*-GlcNAcylation declined markedly as expected and failed to respond to EGF stimulation (Fig. 1*A*). It is known that PKM2 can form active tetramers, less active dimers, or inactive monomers to regulate variable metabolic demands (31, 32, 33). *O*-GlcNAcylation on PKM2 T405/S406 directly destabilized the active tetrameric form and reduced its PK activity (14). When PKM2 *O*-GlcNAcylation was upregulated by EGF, the equilibrium of PKM2 oligomers shifted from tetramers toward dimers and monomers (Fig. 1*B*). Mutating T405/S406 abolished EGF-induced PKM2 detetramerization (Fig. 1*B*), again confirming the important role of T405/S406 *O*-GlcNAcylation in the regulation of oligomeric equilibrium of PKM2. Importantly, the enzymatic activity of PKM2 seemed to follow EGF-mediated alterations in PKM2 *O*-GlcNAcylation and detetramerization. The presence of EGF, which enhanced PKM2 *O*-GlcNAcylation and detetramerization in MCF-7, A549, and U251 cells, reduced the enzymatic activity of WT PKM2 but not the un-*O*-GlcNAcylatable PKM2 T405A/S406A mutant (Fig. 1*C*). Subsequently, we took the advantage of the available PKM2 activator, TEPP-46, which is small molecule capable of stabilizing PKM2 in its tetramer form (34, 35). The addition of TEPP-46 completely blocked EGF-induced reduction in PKM2 activity (Fig. 1*C*). These results showed that EGF-induced upregulation in PKM2 *O*-GlcNAcylation and detetramerization reduced the enzymatic activity of PKM2.

### EGF enhances OGT association with PKM2 to facilitate its *O*-GlcNAcylation

**Figure 2:**
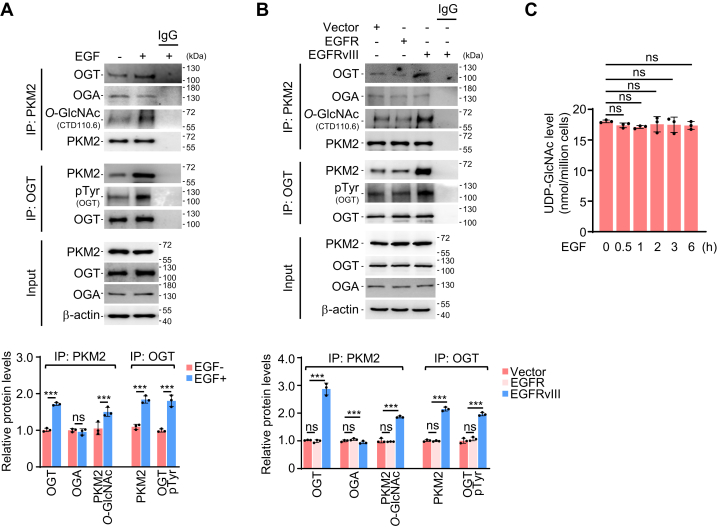
**EGF increases PKM2 association with OGT.***A*, EGF increases the interaction between PKM2 and OGT. (*top*) A549 cells treated by EGF (100 ng/ml for 1 h) were prepared for IP with PKM2 or OGT antibody and analyzed by WB with indicated antibodies. (*bottom*) Relative protein levels of IP. OGT, OGA, and PKM2 *O*-GlcNAc were normalized to immunoprecipitated PKM2, while OGT pTyr and PKM2 were normalized to OGT. Quantification shows mean ± SD (n = 3) with significance determined by student’s *t* test, ∗∗∗*p* < 0.001, ns, nonsignificant. *B*, activated EGFR increases PKM2 binding to OGT. (*top*) In A549 cells deprived for serum and transfected with EGFR or EGFRvIII for 24 h, PKM2- or OGT-associated proteins were obtained from IP and examined by WB with indicated antibodies. (*bottom*) Relative protein levels of IP. OGT, OGA, PKM2 *O*-GlcNAc, OGT pTyr, and PKM2 were normalized to PKM2 or OGT. Quantification shows mean ± SD (n = 3) with significance determined by one-way ANOVA, ∗∗∗*p* < 0.001, ns, nonsignificant. *C*, assay for UDP-GlcNAc level. A549 cells were treated with EGF (100 ng/ml) for different durations and subjected to UDP-GlcNAc analysis by HPLC. Data represent mean ± SD (n = 3) with significance determined by one-way ANOVA, ns, nonsignificant. EGF, epidermal growth factor; EGFR, EGF receptor; OGA, *O*-GlcNAcase; OGT, *O*-GlcNAc transferase.

To understand how PKM2 *O*-GlcNAcylation was regulated by EGF, we examined PKM2 association with OGT and OGA, enzymes which respectively add and remove of *O*-GlcNAc modification on proteins (16, 17). Of note, there are 3 forms of OGT, including ncOGT (nucleocytoplasmic isoform), mOGT (mitochondrial isoform), and sOGT (short isoform), and 2 forms of OGA, namely ncOGA (nucleocytoplasmic isoform) and sOGA (short isoform) (17). Among them, ncOGT and ncOGA are the most universal and dominant ones (17) which we tested in this study. In A549 cells where PKM2 *O*-GlcNAcylation, detetramerization, and activity appeared to be more sensitive to EGF than in the other 2 tested cell lines (Fig. 1), we found that EGF elevated the binding of OGT, but not OGA, to PKM2 (Fig. 2*A*, IP) without influencing the protein levels of OGT and OGA (Fig. 2*A*, Input). Moreover, overexpression of the constitutively active EGFRvIII mutant also led to enhanced OGT association with PKM2 (Fig. 2*B*), arguing that EGF signaling promoted OGT association with PKM2 to stimulate its *O*-GlcNAcylation. In parallel, we also analyzed the level of donor substrate for *O*-GlcNAcylation, UDP-GlcNAc, another determinant for protein *O*-GlcNAcylation. HPLC analysis showed that UDP-GlcNAc levels was unaffected by EGF (Fig. 2*C*). Together, EGF increased OGT association with PKM2 to upregulate PKM2 *O*-GlcNAcylation.

### OGT Y976 phosphorylation induced by EGF is required for OGT-PKM2 interaction

**Figure 3:**
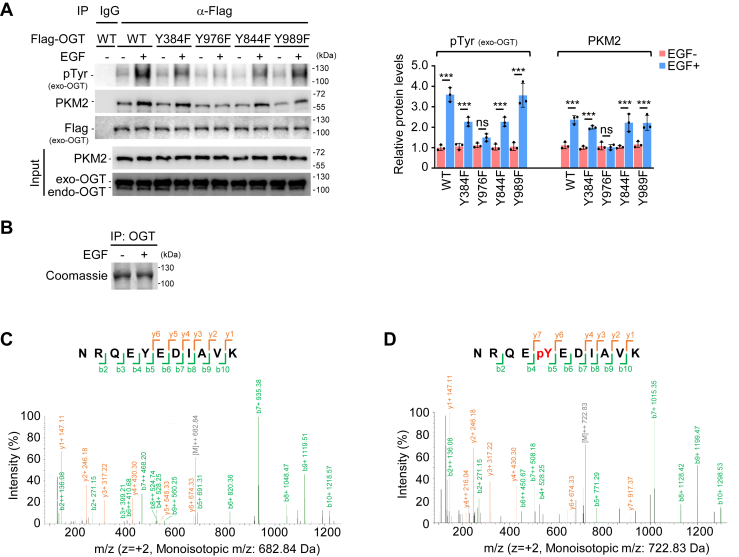
**EGF signal triggers OGT Y976 phosphorylation.***A*, mutation at OGT Y976 abolishes PKM2 binding. (*left*) Transfection of Flag-tagged OGT^WT^, OGT^Y384F^, OGT^Y976F^, OGT^Y844F^, or OGT^Y989F^ along with or without concomitant EGF treatment (100 ng/ml for 1 h) was conducted in A549 cells. OGT-associated proteins were immunoprecipitated and analyzed by WB. Exo, exogenous; endo, endogenous. (*right*) Relative protein levels of IP. OGT pTyr and PKM2 were normalized to Flag (exo-OGT). Quantification shows mean ± SD (n = 3) with significance determined by two-way ANOVA, ∗∗∗*p* < 0.001, ns, nonsignificant. *B–D*, analysis of OGT Y976 phosphorylation by mass spectrometry. A549 cells treated with or without EGF (100 ng/ml) for 1 h were harvested and prepared for IP with OGT antibody prior to mass spectrometry (MS) analysis. *B*, examination of purified OGT proteins. Endogenous OGT were isolated by IP, separated by SDS-PAGE, and stained with Coomassie blue. *C* and *D*, evaluation of OGT phosphorylation. Purified OGT proteins were trypsinized prior to MS analysis. The data were processed with the MASCOT engine, which identified the peptides NRQEYEDIAVK (*C*, EGF untreated, m/z = 682.84 Da) and NRQEpYEDIAVK (*D*, EGF treated, m/z = 722.83 Da). The y and b fragmentations were used to map the phosphorylation site to the Tyr indicated in *red*. EGF, epidermal growth factor; EGFR, EGF receptor; IP, immunoprecipitation; WB, western blotting.

**Figure 4:**
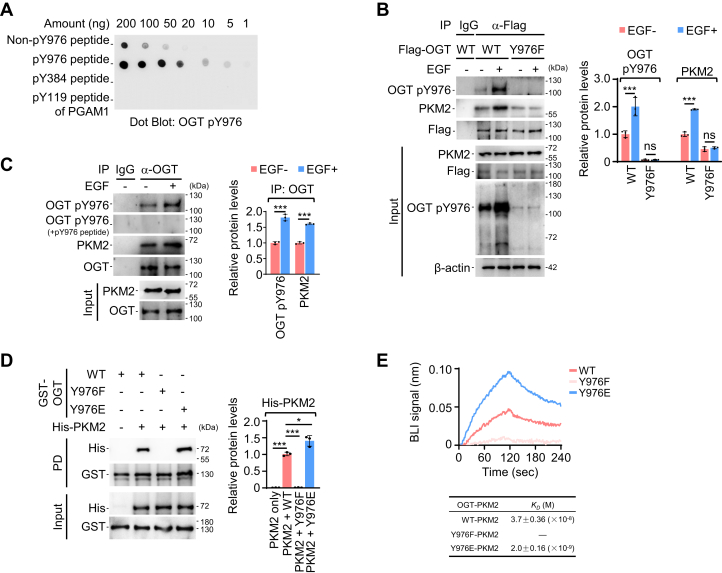
**OGT Y976 phosphorylation is important for PKM2 association.***A*, evaluation of OGT pY976 antibody. Nitrocellulose membrane was spotted with different amounts of unphosphorylated OGT peptide (IAKNRQEYEDIAV), phosphorylated OGT Y976 peptide (IAKNRQEpYEDIAV), phosphorylated OGT Y384 peptide (LQEALMHpYKEAIRI), and phosphorylated phosphoglycerate mutase 1 (PGAM1) Y119 peptide (KIWRRSpYDVP), then probed with OGT pY976 antibody. *B*, examination of Y976 phosphorylation in WT and mutant OGT. (*left*) A549 cells transfected with Flag-tagged OGT^WT^ or OGT^Y976F^ were incubated with or without EGF (100 ng/ml for 1 h) before IP. The phosphorylation site-specific antibody (OGT pY976 antibody) was used for WB analysis. (*right*) Relative protein levels of IP. OGT pY976 and PKM2 were normalized to Flag (OGT). Quantification shows mean ± SD (n = 3) with significance determined by two-way ANOVA, ∗∗∗*p* < 0.001, ns, nonsignificant. *C*, examination of Y976 phosphorylation in endogenous OGT. (*left*) OGT-binding proteins were isolated from A549 cells treated with or without EGF (100 ng/ml) for 1 h by IP and analyzed with WB. OGT pY976 antibody was preincubated with or without phosphorylated OGT Y976 peptide (50 μg/ml) for 1 h before the detection in WB. (*right*) Relative protein levels of IP. OGT pY976 and PKM2 were normalized to OGT. Quantification shows mean ± SD (n = 3) with significance determined by student’s *t* test, ∗∗∗*p* < 0.001. *D*, GST pull-down assay for OGT-PKM2 interaction. (*left*) Recombinant PKM2 and OGT proteins were purified from *E. coli*. GST-tagged OGT^WT^, OGT^Y976F^, and OGT^Y976E^ were immobilized on glutathione agarose beads and mixed with or without His-PKM2 prior to GST pull down (PD). (*right*) Relative protein level of PD. His-PKM2 was normalized to GST-OGT. Quantification shows mean ± SD (n = 3) with significance determined by one-way ANOVA, ∗*p* < 0.05, ∗∗∗*p* < 0.001. *E*, BLI assay for PKM2-OGT interaction. Recombinant His-PKM2 protein was loaded onto Ni-sensor before detection. Subsequently, the sensorgrams for PKM2 binding by GST-tagged OGT^WT^, OGT^Y976F^, and OGT^Y976E^ as well as the associated *K*_*D*_ affinities were obtained. One representative experiment out of three was shown. EGF, epidermal growth factor; EGFR, EGF receptor; IP, immunoprecipitation; OGT, *O*-GlcNAc transferase; WB, western blotting.

Notably, PKM2 is a typical phosphotyrosine-binding protein (20) and OGT contains multiple tyrosine sites, of which Y384 and Y976 in human and Y844 and Y989 in rat were reported to bear phosphorylation (PhosphoSitePlus, https://www.phosphosite.org/uniprotAccAction?id=O15294). Because the phosphotyrosine levels in OGT increased upon EGF stimulation (Fig. 3*A*), we asked whether EGF improved OGT-PKM2 interaction by inducing Y384, Y976, Y844, or/and Y989 phosphorylation. To this end, we generated constructs expressing ncOGT^WT^, ncOGT^Y384F^, ncOGT^Y976F^, ncOGT^Y844F^, and ncOGT^Y989F^ (Fig. S1) and transfected them into A549 cells. Of note, exogenous OGT seemed rather dominant as compared to the endogenous part (Fig. 3*A*, Input). A comparison between WT and mutant OGT showed that mutation at Y976, but not at Y384, Y844, or Y989, inhibited EGF-induced OGT tyrosine phosphorylation (Fig. 3*A*, IP). Consistently, only Y976 mutation blocked EGF-induced interaction between OGT and PKM2 (Fig. 3*A*). Our mass spectrometry (MS) analysis for OGT immunoprecipitated from A549 cells further confirmed the phosphorylation of OGT at Y976 (Fig. 3, *B*–*D*). To precisely investigate the function of OGT Y976 phosphorylation, we next generated an antibody that can specifically recognize OGT Y976 phosphorylation (Fig. 4*A*). Notably, neither the irrelevant phosphopeptide from OGT nor a random phosphopeptide from an unrelated protein was recognized by this antibody (Fig. 4*A*). Although certain unspecific and weak recognitions appeared in the western blotting for total cell lysates (Fig. 4*B*, Input), the newly generated antibody could specifically detect OGT Y976 phosphorylation in our immunoprecipitations (Fig. 4, *B* and *C*). A complete blockade of OGT Y976 phosphorylation detection by pY976 peptide further confirmed the specificity of the new antibody (Fig. 4*C*). Importantly, we observed that EGF stimulation evidently upregulated OGT Y976 phosphorylation (Fig. 4, *B* and *C*). When Y976 was mutated to F, this EGF-induced upregulation was completely abolished (Fig. 4*B*). Moreover, using GST pull down and biolayer interferometry (BLI) with recombinant OGT and PKM2 purified from *Escherichia coli*, we showed that OGT association with PKM2 was disrupted by the Y976F mutation but was enhanced while Y976 was replaced by glutamate (Y976E) (Figs. 4, *D* and *E* and S1) that mimics constitutive phosphorylation (36, 37, 38). These observations confirmed the important role of Y976 phosphorylation in OGT-PKM2 interaction.

**Figure 5:**
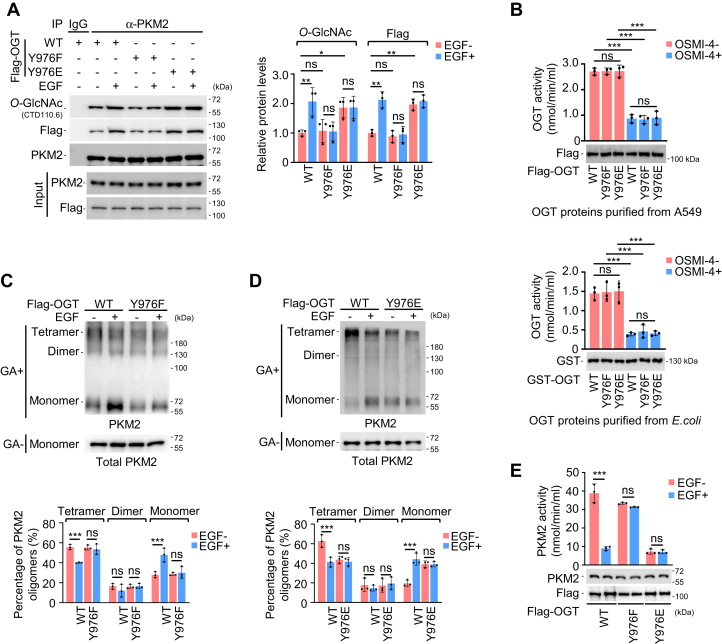
**OGT Y976 phosphorylation is important for EGF-induced *O*-GlcNAcylation on PKM2.***A*, impact from mutations in OGT on PKM2 *O*-GlcNAcylation. (*left*) A549 cells transfected with Flag-tagged OGT^WT^, OGT^Y976F^ (unphosphorylatable), or OGT^Y976E^ (unphosphorylatable) were incubated with or without EGF (100 ng/ml) for 1 h and then subjected to IP analysis for OGT-PKM2 interaction and PKM2 *O*-GlcNAcylation. (*right*) Relative protein levels of IP. PKM2 *O*-GlcNAc and Flag-OGT were normalized to PKM2. Quantification shows mean ± SD (n = 3) with significance determined by two-way ANOVA, ∗*p* < 0.05, ∗∗*p* < 0.01, ns, nonsignificant. *B*, activity assay for OGT. Flag-tagged OGT^WT^, OGT^Y976F^, and OGT^Y976E^ proteins purified from A549 cells (*top*), and GST-tagged OGT proteins purified from *E. coli* (*bottom*) were evaluated for their enzymatic activity in the presence or absence of OSMI-4 (3 μM). Reduction in UDP-GlcNAc is being measured and calculated to indicate OGT activity. Data represent mean ± SD (n = 3) with significance determined by two-way ANOVA, ns, nonsignificant. *C* and *D*, assay for PKM2 oligomers with OGT mutants. *(top)* The whole cell lysates from A549 cells treated as (*A*) were crosslinked and analyzed by WB. (*bottom*) Quantification of PKM2 tetramers, dimers, and monomers was based on the normalization to total PKM2. Data represent mean ± SD (n = 3) with significance determined by two-way ANOVA, ∗∗∗*p* < 0.001, ns, nonsignificant. *E*, Assay for PKM2 activity with OGT mutants. PKM2 isolated from cells treated as (*A*) were assayed for enzymatic activity. Data represent mean ± SD (n = 3) with significance determined by two-way ANOVA, ∗∗∗*p* < 0.001, ns, nonsignificant. EGF, epidermal growth factor; IP, immunoprecipitation; OGT, *O*-GlcNAc transferase.

Furthermore, mutating OGT Y976 to F compromised EGF-induced upregulation in PKM2 *O*-GlcNAcylation (Fig. 5*A*). However, when OGT Y976 was replaced by E, the level of PKM2 *O*-GlcNAcylation was elevated, irrespective of the presence of EGF (Figs. 5*A* and S1). To check whether changes in PKM2 *O*-GlcNAcylation were due to altered OGT activity upon point mutations, we took advantage of the published method (39, 40) with a few modifications implemented to avoid unexpected interferences and used HPLC to measure UDP-GlcNAc (Fig. 5*B*), the donor molecule for *O*-GlcNAcylation mediated by OGT. In our assay, OGT inhibitor OSMI-4 (41) reduced OGT activity as expected (Fig. 5*B*), while different mutations failed to affect UDP-GlcNAc levels in the absence or presence of OSMI-4 (Fig. 5*B*). Although this assay does not discriminate between OGT transferring GlcNAc to a peptide or water (hydrolysis), the WT and mutant OGTs had similar activity in this assay (Fig. 5*B*). In addition, mutation of OGT Y976F blocked EGF-induced detetramerization and reduction in PKM2 activity, while OGT Y976E mutant maintained PKM2 detetramerization and reduction in PKM2 activity (Fig. 5, *C*–*E*). Collectively, we conclude that OGT Y976 phosphorylation played an essential role in EGF-dependent regulation of PKM2.

### Y976 phosphorylation might be important for OGT recognition by phosphotyrosine-binding proteins

**Figure 6:**
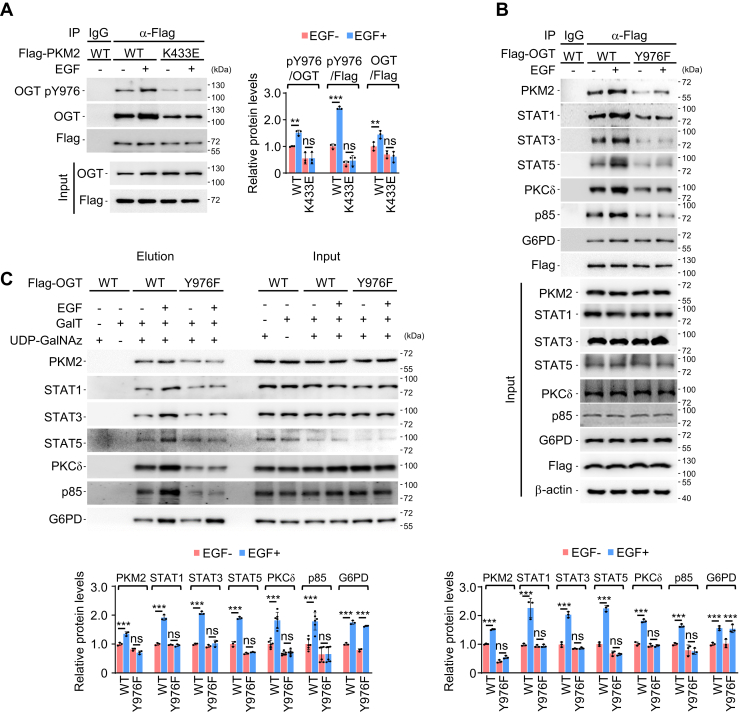
**OGT Y976-phosphorylation is important for the association of representative phosphotyrosine-binding proteins.***A*, PKM2 K433 is required for OGT binding. *(left)* PKM2-associated proteins immunoprecipitated from A549 cells were analyzed by WB with indicated antibodies. (*right*) Relative protein levels of IP. pY976 and OGT was normalized to OGT and/or Flag (PKM2). Quantification shows mean ± SD (n = 3) with significance determined by two-way ANOVA, ∗∗*p* < 0.01, ∗∗∗*p* < 0.001, ns, nonsignificant. *B*, mutation at Y976 disrupts OGT interaction with selected phosphotyrosine-binding proteins. A549 cells transfected with Flag-tagged OGT^WT^ or OGT^Y976F^ were incubated with or without EGF (100 ng/ml) for 1 h. (*top*) The whole lysates and OGT-associated proteins from IP were analyzed by WB. (*bottom*) Relative protein levels of IP. Indicated proteins were normalized to Flag-OGT. Quantification shows mean ± SD (n = 3) with significance determined by two-way ANOVA, ∗∗∗*p* < 0.001, ns, nonsignificant. *C*, mutation at OGT Y976 impairs *O*-GlcNAcylation on selected phosphotyrosine-binding proteins. (*top*) A549 cells treated and transfected as in (*B*) were subjected to Click-iT *O*-GlcNAc Enzymatic Labeling System. *O*-GlcNAcylated proteins were biotinylated, precipitated, and analyzed by WB. (*bottom*) Relative protein levels of elution. *O*-GlcNAcylation level for each protein (elution) was normalized to its total protein (input). Quantification shows mean ± SD (n ≥ 3) with significance determined by two-way ANOVA, ∗∗∗*p* < 0.001, ns, nonsignificant. EGF, epidermal growth factor; EGFR, IP, immunoprecipitation; WB, western blotting; OGT, *O*-GlcNAc transferase.

As a phosphotyrosine-binding protein, PKM2 recognizes and binds to various targets, such as Src and PDGFR, which all contain phosphorylated tyrosine residues (20). A positive charged amino acid K433 in PKM2 has been shown to be important for PKM2 association with phosphotyrosine-containing proteins (20). As anticipated, mutation at PKM2 K433 also impaired PKM2 interaction with OGT upon EGF stimulation (Fig. 6*A*). Y976 phosphorylation of OGT that associated with PKM2 K433E was indeed lower than that in PKM2 WT-bound OGT (Fig. 6*A*). These observations corroborated the important role of K433 in bridging PKM2 interaction with phosphotyrosine-containing proteins. Notably, the disruption of PKM2 association with OGT upon PKM2 K433 mutation was incomplete, indicating additional mechanism(s) might be also involved in the regulation of PKM2-OGT interaction. Other than PKM2, a number of phosphotyrosine-binding proteins have also been found to be *O*-GlcNAcylated (20, 21, 22, 23, 24, 25, 26, 27). Given the critical role of Y976 phosphorylation in EGF-induced OGT interaction with PKM2 (Figs. 3 and 4), we asked whether this PTM is also important for OGT recognition by a broader range of phosphotyrosine-binding proteins.

To this end, phosphotyrosine-binding proteins that bear *O*-GlcNAcylation, including transcriptional factor STAT1, STAT3, STAT5, protein kinase PKCδ, and PI3K subunit p85 (21, 22, 23, 24, 25, 26, 27), were selected for the interaction assay with OGT. While EGF did not affect the protein levels of selected factors regardless of the presence of either OGT^WT^ or OGT^Y976F^, binding of STAT1, STAT3, STAT5, PKCδ, and p85 to OGT was upregulated by EGF treatment (Fig. 6*B*). Importantly, EGF-mediated association with OGT for these factors was substantially compromised when Y976 in OGT was mutated (Fig. 6*B*). This argued that, in addition to PKM2, other phosphotyrosine-binding proteins might also need Y976 phosphorylation to interact with OGT. Of note, glucose-6-phosphate dehydrogenase (G6PD), which is a known *O*-GlcNAcylated protein regulated by OGT, but not a phosphotyrosine-binding protein, was included in our interaction assay as a negative control. It appeared that G6PD-OGT interaction was not affected by Y976 phosphorylation (Fig. 6*B*). Furthermore, in the presence of EGF, OGT^WT^, instead of OGT^Y976F^ mutant, enhanced the *O*-GlcNAcylation of STAT1, STAT3, STAT5, PKCδ, and p85 (Fig. 6*C*). Taken together, these results suggest that Y976 phosphorylation is important for OGT interaction with phosphotyrosine-binding proteins.

## Discussion

**Figure 7:**
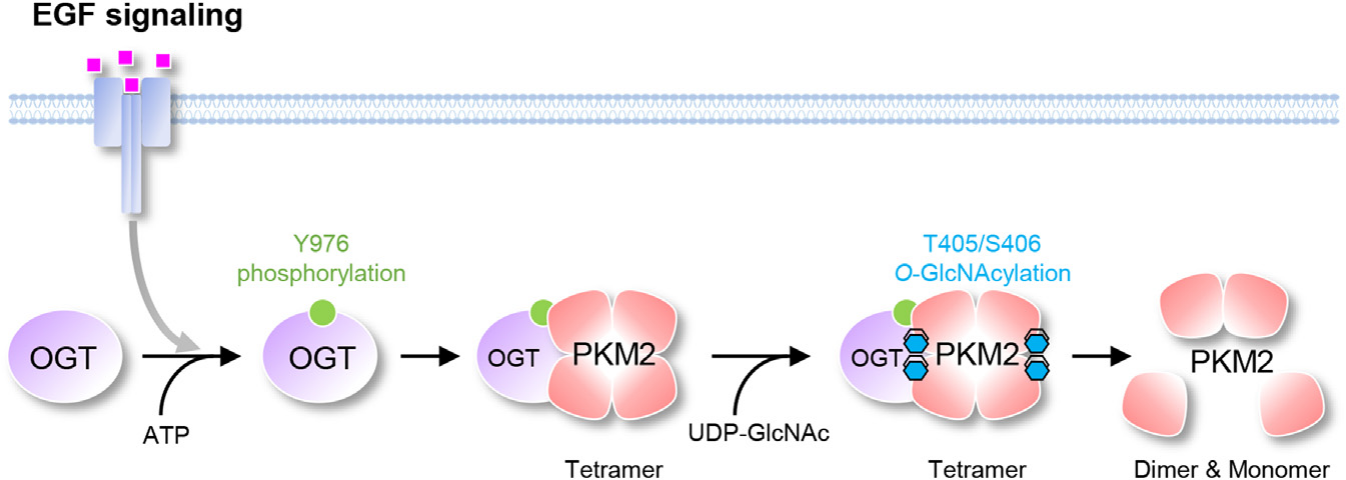
**Model of PKM2 regulation by OGT upon EGF stimulation.** EGF stimulates the phosphorylation of OGT at Y976 to promote its association with PKM2. As a consequence, PKM2 are *O*-GlcNAcylated at T405/S406, resulting in the disassembly of active tetramers into less active dimers and monomers. EGF, epidermal growth factor; OGT, *O*-GlcNAc transferase.

In this study, we have uncovered a mechanism by which EGF regulates PKM2 function. By mediating OGT phosphorylation at Y976, EGF signaling stimulates OGT interaction with PKM2 and consequently promoted PKM2 *O*-GlcNAcylation and detetramerization (Fig. 7).

As a key enzyme regulating glycolysis, PKM2 is highly *O*-GlcNAcylated in various types of tumor cells and tissues and involved in the regulation of the Warburg effect (14). We previously showed that, on one hand, *O*-GlcNAcylation-dependent PKM2 detetramerization resulted in reduced PK activity, leading to the rewiring of metabolic fluxes toward anabolic pathways for rapid cell proliferation (14). On the other hand, destabilized PKM2 tetramers facilitated their nuclear translocation and in turn stimulated GLUT1 and LDHA expression to promote the Warburg effect (14). EGF-mediated PKM2 *O*-GlcNAcylation and detetramerization are likely to influence cell proliferation by modulating both metabolic and nuclear functions of PKM2. The identification of Y976 phosphorylation coupled with EGF signaling may pave the way for limiting uncontrolled cancer cell proliferation as well as tumor growth by blocking PKM2 functions. Meanwhile, we also notice that the abolishment of OGT Y976 phosphorylation in OGT^Y976F^ did not seem to jeopardize PKM2 *O*-GlcNAcylation, detetramerization, or activity under normal condition without EGF (Fig. 5), implying that additional mechanism(s) may be involved in the control of PKM2 function, especially under an unperturbed situation.

*O*-GlcNAcylation is a noncanonical and reversible glycosylation, which involves the attachment of single *O*-linked GlcNAc moieties to Ser and Thr residues (16, 17). Dynamic *O*-GlcNAc cycling regulates a wide variety of cellular processes involving gene transcription, signal transduction, and metabolism (16, 17, 42). The donor substrate, UDP-GlcNAc, is the end product of nutrient fluxes and synthesized through the hexosamine biosynthetic pathway, which integrates glucose, amino acid, fatty acid, and nucleotide metabolism (16). Obviously, *O*-GlcNAcylation on proteins that is recognized as a “nutrient sensor” is tightly linked with *O*-GlcNAc level as well as nutrient availability. More recent studies revealed that *O*-GlcNAcylation is also responsive to different environmental stimuli, such as heat shock, hypoxia, cytokines, and growth factors (17). Whether the mechanism for the regulation of PKM2 by OGT Y976 phosphorylation could be useful for cells to respond to or cope with other types of stimuli, in addition to EGF, remains to be tested.

Hitherto, thousands of proteins, including transcription factors, signal proteins, and metabolic enzymes, have been found to be *O*-GlcNAcylated (17). Disturbed *O*-GlcNAc homeostasis and aberrant *O*-GlcNAcylation are frequently associated with diabetes, neurodegeneration, and cancers (17). How OGT recognizes and selects substrate proteins for *O*-GlcNAcylation has been a long-standing question in the field. Albeit identification of consensus sequences from substrate proteins for OGT recognition has not been successful, accumulating attempts based biochemical, biophysical, and computational analyses of the molecular structures of OGT have suggested different scenarios (17): (i) distinct adapter proteins that recruit different substrates to OGT in a context-dependent manner probably play an important role in OGT substrate selection (17); (ii) the N-terminal tetratricopeptide repeat domain in OGT, which is an extended superhelical structure composed of up to 13.5 tetratricopeptide repeats, has been reported to function as a scaffold for the recognition and assembly of distinct protein complexes (43); (iii) a possibility that OGT nonspecifically *O*-GlcNAcylates proteins in their unstructured/flexible regions (loops or termini) has also been proposed (43). Apparently, each of these mechanisms can only explain some of the available cases. A comprehensive understanding for the control of OGT substrate selection is still lacking. We here demonstrated that EGF-mediated phosphorylation of OGT at Y976 navigated the association of PKM2 with OGT. Also, the regulation of OGT recognition by Y976 phosphorylation appeared to be important for the association of a set of phosphotyrosine-binding proteins as well, indicating that OGT Y976 phosphorylation is a special PTM with important function in substrate recognition. How PTMs on OGT regulate substrate recognition is probably an attractive area of future investigations.

By stimulating OGT Y976 phosphorylation, EGF improved OGT-PKM2 interaction and subsequently enhanced PKM2 *O*-GlcNAcylation and detetramerization. These findings reveal *O*-GlcNAcylation as a link integrating EGF signaling with metabolic regulation and shed lights on the exploitation of *O*-GlcNAcylation in therapeutic treatment against EGF receptor expressing cancers.

## Experimental procedures

### Cell lines and reagents

Human MCF-7 breast cancer cells, A549 lung cancer cells, and U251 glioblastoma cells were cultured in Dulbecco's modified Eagle's medium (Sigma–Aldrich), supplemented with 10% fetal bovine serum and 1% penicillin/streptomycin. All cell lines aforementioned were purchased from the Cell Bank of Type Culture Collection of Chinese Academy of Science. EGF was purchased from R&D Systems. TEPP-46 and OSMI-4 was purchased from MedChemExpress.

### DNA constructs and mutagenesis

PCR-amplified human PKM2 complementary DNA was cloned into p3 × Flag-CMV-10 or pET-28a vector. Original ncOGT plasmid was generously provided by Dr Gerald W. Hart (School of Medicine, Johns Hopkins University) (44). We subsequently constructed ncOGT into p3 × Flag-CMV-10 (Flag-tag) and pGEX-4T-2 (GST-tag) vectors (Fig. S1). Mutations, including PKM2^T405A/S406A^, PKM2^K433E^, OGT^Y384F^, OGT^Y976F^, OGT^Y844F^, OGT^Y989F^, and OGT^Y976E^ were made using the QuickChange Site-directed Mutagenesis Kit (TransGen Biotech). Transient transfection of DNA constructs was performed using Lipofectamine 2000 reagents (Thermo) according to the vendor’s instructions.

### Immunoprecipitation and Western blotting

Cells were lysed in cold Western blotting (WB)-immunoprecipitation (IP) lysis buffer (Beyotime) for 30 min and centrifuged (4 °C, 5 min at 14,000*g*) to remove cell debris. Supernatants were collected and used as the whole cell lysates. Flag-PKM2 protein were immunoprecipitated from the whole cell lysates using anti-Flag antibody coupling to Protein A/G PLUS Agarose beads (Santa Cruz Biotechnology) for 3 h at 4 °C. Nonspecific mouse IgG antibody (Boster) coupling to Protein A/G PLUS Agarose beads was used as a negative control. Immunoprecipitated proteins on the beads were collected in 2 ways: (1) directly boiled off with loading buffer (100 mM Tris–Hcl, pH6.8, 4% SDS, 0.2% bromophenol orchid, 20% glycerine, 200 mM β-mercaptoethanol) for 5 min; (2) competitively eluted by poly DYKDDDDK (Flag) peptide (400 μg/ml) for 1 h at 4 °C. The IP samples were analyzed by SDS-PAGE (6%∼8% separation gel) in WB.

Antibodies used in this study include PKM2 (CST, #4053S), OGT (CST, #24083), OGA (Abcam, #ab124807), pTyr (CST, #8954), Flag (Sigma, #F1804), β-actin (Sigma, #A4700), STAT1 (SAB, College Park, #41462), STAT3 (CST, #30835), STAT5 (SAB, #41466), PKCδ (Abclonal, #A0471), p85 (Abclonal, #A11526), G6PD (SAB, #32301), and *O*-GlcNAcylation (CTD110.6, Sigma, #O7764; RL2, Abcam, #ab2739). OGT pY976 site-specific antibody was generated at Shanghai Genomic Inc. The OGT phosphopeptide C-IAKNRQEY(p)EDIAV and nonphosphopeptide C-IAKNRQEYEDIAV were synthesized and conjugated with keyhole limpet hemocyanin, respectively. The serum antibody was produced by immunizing rabbits with synthetic phosphopeptide IAKNRQEY(p)EDIAV after 5 immunizations. Nonphospho-specific antibodies were removed by chromatography using nonphosphopeptide IAKNRQEYEDIAV. OGT pY976 site-specific antibody was purified by affinity chromatography using epitope-specific IAKNRQEY(p)EDIAV phosphopeptide. In following validation assays, we have thoroughly verified the specificity of OGT pY976 polyclonal antibody.

### GST pulldown assay

*E*. *coli* cells transfected with GST-OGT or His-PKM2 were induced by IPTG (1 mM) at 16 °C for 24 h. GST-OGT fusion proteins immobilized on Glutathione Sepharose 4B (GE Healthcare) were mixed with or without His-PKM2 fusion proteins rolling at 4 °C overnight in lysis buffer containing 50 mM Tris–HCl, pH 8.0, 120 mM NaCl, 1 mM DTT, 10% glycerol, and plus 1 mM PMSF. The agarose resin was washed extensively with lysis buffer before eluting with lysis buffer (pH 7.5) containing 20 mM glutathione. Eluted proteins were analyzed by WB.

### BLI

The BLI assay was performed on Octet RED96 System (Pall ForteBio). His-tagged PKM2 (50 μg/ml) purified from *E. coli* were immobilized onto a Ni-NTA biosensor (Pall ForteBio) that had been equilibrated in running buffer containing 20 mM Hepes (pH 7.5), 150 mM NaCl, and 0.02% Tween-20 for 3 min. Subsequently, the biosensor coated with His-PKM2 was transferred into GST-tag or GST-OGT containing buffer or analyte-free buffer for the detection of PKM2-OGT association. Binding constants were determined using Octet System Data Analysis software 8.11 (Pall ForteBio).

### PKM2 oligomerization assay

The whole cell lysates (4 mg/ml) were crosslinked with 0.025% glutaraldehyde for 3 min at 37 °C and terminated with Tris–HCl (pH 8.0, 50 mM). Subsequently, samples were analyzed by WB with indicated antibodies.

### PKM2 activity assay

PKM2 proteins immunoprecipitated from the whole cell lysates were incubated with reaction buffer (30 μM pyruvate, 6.6 μM NADH, 0.2 M Tris–HCl, 500 μM FBP, pH 7.3) for 30 min at room temperature (RT). PK activity was then measured with a colorimetric based PK activity assay kit (Sigma) according to the manufacturer’s protocol.

### HPLC assay for UDP-GlcNAc

About 5 × 10^6^ cells were resuspended in 500 μl methanol (80%, −80 °C precold) and incubated for 20 min at −80 °C, centrifuged for about 40 min, 12000*g* to remove the sediment, then subjected to detect the UDP-GlcNAc level by HPLC (LC-16, Shimadzu). In brief, samples and a series of standard UDP-GlcNAc (Sigma) solutions were filtered at 0.22 μm. Ten microliter aliquots of samples were directly injected into HPLC C18 column (250 mm × 4.6 cm, 5 μm, 100 Å, Kromasil), which is suitable to separate the nucleotide sugars (45), while a pump system supplied moving phase A (0.1% trifluoroacetic acid [TFA] in distilled water) and moving phase B (0.1% TFA in acetonitrile [ACN]). Absorbance measurements were made at 214 nm on a UV-detector (SPD-10A, Shimadzu). The profile of the gradient moving phase was as follows: 0∼3 min, 90% A, 10% B, and the flow rate was 1 ml/min. The level of UDP-GlcNAc was calculated based on the peak area referenced to standards.

### OGT activity assay

OGT activity assay was modified from the previously published protocol (39, 40). According to the established reaction system, OGT use UDP-GlcNAc as substrates to modify YSDSPSTST peptides and release UDP, which combine with phosphoenolpyruvate to support PKM2-mediated pyruvate formation. The previous protocol analyzed pyruvate concentration to evaluate the catalytic activity of OGT. Because OGT can directly target PKM2 and regulate its functions, we decided to avoid the potential interference from PKM2 during the assessment of OGT activity. In the modified assay, we only removed PKM2 and phosphoenolpyruvate from the reaction system and kept the rest of the settings in the exact same way. By using HPLC, we followed the level of UDP-GlcNAc to monitor OGT activity. In brief, OGT proteins (300 nM) were mixed with UDP-GlcNAc (1 mM) and YSDSPSTST peptide (200 μM) in reaction buffer (50 mM Tris–HCl, pH 7.4, 1 mM DTT, 10 mM KCl, and 12.5 mM MgCl_2_) in the presence or absence of OSMI-4 (3 μM), then incubated at 37 °C for 1 h. The reaction was terminated by adding methanol solution of the same volume. The supernatant was centrifuged for about 40 min, 12,000*g* to remove the sediment, filtered at 0.22 μm, and then subjected to detect the UDP-GlcNAc level by HPLC. The reduction of UDP-GlcNAc in the reaction indicates changes in OGT activity.

### MS analysis for OGT phosphorylation

In A549 cells (5 × 10^8^ cells) pretreated with or without EGF (100 ng/ml) for 1 h respectively, endogenous OGT proteins were isolated by IP followed by SDS-PAGE and subjected to in-gel protein digestion and peptide recovery. The operation steps are as follows: cut 3 excised gel slices from each gel into 1 mm^3^ cubes, add 500 μl of 50 mM ammonium bicarbonate/ACN (1:1, v/v) solution, and wash until Coomassie blue disappear. Remove the supernatant, add 500 μl of ACN, and incubate for 10 min. Remove the ACN, rehydrate the gel slices in 10 mM DTT/50 mM ammonium bicarbonate to completely cover the gel slices, and incubate at 56 °C for 1 h. Remove the supernatant, add 500 μl of ACN, and incubate for 10 min. Remove the ACN and add the 50 mM iodoacetamide/50 mM ammonium bicarbonate to completely cover the gel slices. Incubate for 60 min at RT in the dark. Remove the iodoacetamide/ammonium bicarbonate, add 500 μl of ACN, and incubate for 10 min. Remove the ACN solution and add just enough enzyme digestion solution to cover the gel slices. Incubate the gel pieces on ice for 45 min. Add 10 μl of enzyme digestion solution to keep the gel pieces wet during enzymatic digestion. Incubate overnight at 37 °C. Add 100 μl extraction solution (5% TFA-50% ACN-45% ddH_2_O) at 37 °C water bath for 1 h, sonicate, centrifuge, and then transfer the extract to a fresh microcentrifuge tube. Lyophilize the extracted peptides to near dryness. Resuspend peptides in 10 μl of 0.1% formic acid.

The solutions containing peptides released during in-gel digestion were measured using Nanoflow UPLC (Ultimate 3000 system, Thermo) coupled to a mass spectrometer (Q Exactive Hybrid Quadrupole-Orbitrap, Thermo). Briefly, the trypsinized peptides were firstly trapped and desalted on an Acclaim PepMap100 C18 Nano-Trap Column (75 μm × 2 cm, 3 μm, 100 Å, Thermo) with a pump system supplied moving phase A (0.1% formic acid in distilled water) and moving phase B (0.1% formic acid in ACN). The profile of the gradient moving phase was as follows: 0∼4 min, 5% B; 4∼30 min, 5% to 40% B; 30∼35 min, 40% to 80% B; 35∼45 min, 80% B; 45 to 45.1 min, 5% B; 45.1 to 60 min, 5% B; and the flow rate was 0.4 μl/min. The peptides were then reverse eluted and loaded into the analytical capillary C18 column (Venusil × BPC, 75 μm × 10 cm, 5 μm, 300 Å, Agela Technologies) connected inline to the mass spectrometer. Different fractions of the eluate were injected into the Q-Exactive MS set in a positive ion mode and the data-dependent manner with a full MS scan from 350 to 2000 m/z. High collision energy dissociation was employed as the MS/MS acquisition method. Raw MS/MS data were converted into an MGF format using Proteome Discoverer 1.2 (Thermo). The exported MGF files were searched with Mascot v2.3.01 against the human OGT database (UniProt_O15294; 1 sequence; 1046 residues) with a tryptic specificity allowing 2 missed cleavage. The sequences coverage is ∼90%. Carbamidomethylation (C) was considered as fixed modification, whereas oxidation (M) and Gln->pyro-Glu (N-term Q) as variable modifications. The mass tolerance for MS and MS/MS was 15 ppm and 20 mmu, respectively. Proteins with false discovery rates <0.05 were further analyzed. The result was filtered by score >25. The MS proteomics data have been deposited to the ProteomeXchange Consortium *via* the PRIDE (46) partner repository with the dataset identifier PXD031593.

### Click-iT *O*-GlcNAc enzymatic labeling for the detection of *O*-GlcNAcylation

Chemoenzymatic labeling and biotinylation of proteins in total cell lysates were carried out as described previously (14, 47). In brief, proteins (200 μg) were labeled utilizing the Click-iT *O*-GlcNAc Enzymatic Labeling System (Invitrogen). The permissive mutant β-1,4-galactosyltransferase (GalT) is responsible for the transfer of azido-modified galactose (GalNAz) from UDP-GalNAz to *O*-GlcNAc residues on target proteins. Modified proteins were detected utilizing the Click-iT Biotin Protein Analysis Detection Kit protocol (Invitrogen). Biotinylated proteins were resolubilized in binding buffer (0.1 M phosphate, 0.15 M NaCl, 0.1% SDS, 1% NP-40, pH 7.2). Appropriate amount of streptavidin resin (Thermo) was added to incubate with the mixture overnight at 4 °C. The streptavidin-bound complex was washed with binding buffer. Following the removal of supernatants, pellets were eluted by boiling with loading buffer (2% SDS, 10% glycerol, 2.5% 2-mercaptoethanol, 62.5 mM Tris–HCl, pH 6.8) and analyzed by WB.

### Statistical analysis

All experiments were done at least 3 times independently. Data were analyzed using Prism Version 8 software (GraphPad). For data with 2 groups, unpaired *t* tests were performed. For datasets with 3 or more groups, a one-way or two-way ANOVA was performed followed by Tukey’s multiple comparison test. Data are presented as the mean ± SD values. Calculated *p* values are indicated on individual figures.

## Data availability

All data are contained within the article.

## Supporting information

This article contains supporting information.

## Conflict of interest

The authors declare that they have no conflicts of interest with the contents of this article.
