# Supplementary material for: EGF promotes PKM2 O-GlcNAcylation by stimulating O-GlcNAc transferase phosphorylation at Y976 and their subsequent association
Source: J Biol Chem. 2022 Aug 3;298(9):102340. doi: 10.1016/j.jbc.2022.102340 (PMC9436816; doi:10.1016/j.jbc.2022.102340)
Supplement: Supplemental Figure S1 [file mmc1.pdf]

Figure S1

A

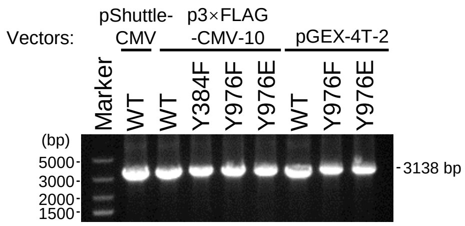

B

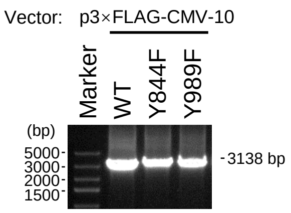

Fig S1. Confirmation of DNA constructs expressing ncOGT.

(A,B) PCR amplification of the DNA sequence encoding wild-type (WT) or mutant nucleocytoplasmic isoform of OGT (ncOGT) in pShuttle-CMV, p3×FLAG-CMV-10 (Flag-tag) and pGEX-4T-2 (GST-tag) vectors, respectively. Forward primer 5'-ATGGCGTCTTCCGTGGGCAAC-3'; Reverse primer 5'-TTATGCTGACTCAGTGACTTC-3'.
